# Role of Wnt5a in modulation of osteoporotic adipose‐derived stem cells and osteogenesis

**DOI:** 10.1111/cpr.13747

**Published:** 2024-09-17

**Authors:** Lin Liu, Shihong Luo, Qiumei Li, Kui Huang, Yuan Jiang, Lu Zeng, Xiaorong Lan, Qing Li, Jingang Xiao

**Affiliations:** ^1^ Department of Oral and Maxillofacial Surgery, The Affiliated Stomatological Hospital Southwest Medical University Luzhou China; ^2^ Luzhou Key Laboratory of Oral & Maxillofacial Reconstruction and Regeneration Luzhou China; ^3^ Department of Oral Implantology, The Affiliated Stomatological Hospital Southwest Medical University Luzhou China; ^4^ Medical Service Center of Sichuan Province Chengdu China; ^5^ Department of Oral and Maxillofacial Surgery The Affiliated Hospital of Southwest Medical University Luzhou China

## Abstract

Osteoporosis, a condition marked by the deterioration of bone microarchitecture and increased facture risk, arises from a disruption in bone metabolism, with osteoclasts surpassing osteoblasts in bone resorption versus formation. The Wnt signalling pathway, a key regulator of bone maintenance, remains partially understood in osteoporosis. Our research delves into the role of Wnt‐related molecules in this disease. In osteoporotic adipose‐derived stem cells (OP‐ASCs), we detected a significant decrease in *Ctnnb1* and *Frizzled‐6* (*Fzd6*), contrasted by an increase in *Gsk‐3β* and *Wnt5a*. Activation of the Wnt pathway by LiCl resulted in elevated *Ctnnb1* and *Fzd6*, but decreased *Gsk‐3β* and *Wnt5a* levels, promoting OP‐ASCs' bone‐formation capacity. In contrast, inhibition of this pathway by DKK‐1 led to diminished *Ctnnb1* and *Fzd6*, and increased *Gsk‐3β* and *Wnt5a*, adversely affecting osteogenesis. Furthermore, our findings show that overexpressing *Wnt5a* impedes, while silencing it enhances the bone‐forming capability of OP‐ASCs. In a cranial bone defect model, the implantation of *Wnt5a*‐silenced OP‐ASCs with biphasic calcium phosphate scaffolds significantly promoted new bone formation. These observations indicated a repression of the canonical Wnt pathway and a stimulation of the non‐canonical pathway in OP‐ASCs. Silencing *Wnt5a* increased the osteogenic and regenerative abilities of OP‐ASCs. Our study suggests targeting Wnt5a could be a promising strategy for enhancing bone regeneration in post‐menopausal osteoporosis.

## Graphical abstract

In this study, OP‐ASCs were successfully isolated and cultured from the inguinal fat of the OP mouse with bilateral ovariotomy (OVX). Activation of the canonical Wnt signalling pathway markedly enhanced osteogenesis in OP‐ASCs, concurrently suppressing Wnt5a and GSK‐3β expression. Lentiviral‐mediated silenced of *Wnt5a* could enhance the osteogenic ability of OP‐ASCs by activating the canonical Wnt signalling pathway to promote the repair of critical‐sized calvarial defects in osteoporotic mice.

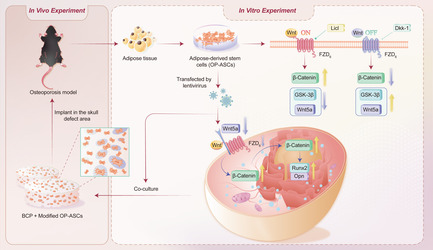

## 1 INTRODUCTION

Osteoporosis (OP) is a prevalent skeletal disorder resulting from the dysregulation of osteoclast and osteoblast activities, resulting in a greater propensity for bone resorption than for bone formation, and is closely linked to post‐menopausal oestrogen deficiency, aging or diabetes.^1^, ^2^, ^3^ Characterized by reduced bone density and mass, OP leads to bone microarchitecture deterioration and increased fractures risk.^4^, ^5^ Fractures can cause severe pain and disability, particularly in the spine and hip, impacting quality of life and posing life‐threatening risks.^6^ Approximately 20% of individuals with osteoporotic hip fractures will die within 1 year.^7^ Current clinical treatments for OP primarily involves anti‐bone resorptive and bone anabolic drugs, which necessitate long‐term use and may lead to various adverse reactions like gastrointestinal irritation, venous embolism, atypical femoral fractures, osteonecrosis of the jaw and potentially even breast cancer.^8^, ^9^ Surgical intervention is the main approach for treating osteoporotic fractures, but it carries potential risks of nerve damage and recurrent fractures.^10^ With the aging population, the incidence of OP is increasing dramatically and it is expected that there will be over 200 million osteoporosis patients by 2050.^11^ This highlights the urgent need for effective treatment addressing fracture healing delays, bone fragility and osteoporotic bone defects caused by osteoporosis.

Accumulating researches have shown that stem cells have the advantage of remarkable regenerative capacity, lower adverse effects and personalized treatment modalities.^12^ Therapies using stem cells for bone defect restoration show great prospects for clinical applications.^13^ Adipose‐derived stem cells (ASCs) are readily obtainable through minimally invasive methods and are known for their high proliferation rate and efficacy in bone tissue repair and regeneration.^14^, ^15^ It has been noted that although ASCs derived from osteoporotic mice (OP‐ASCs) have the potential for osteogenic differentiation, their osteogenic differentiation capacity is lower than that of normal ASCs, resulting in a substantial reduction in the ability to facilitate the deposition of minerals.^16^ Identifying the specific factors impeding the bone‐forming capabilities of OP‐ASCs could pave the way for effective OP‐ACSs based cellular therapies.

Recent studies have underscored the Wnt signalling pathway's central role in OP development and its crucial function in maintaining bone homeostasis and regulating mesenchymal stem cells differentiation into obsteoblasts.^17^, ^18^ The canonical Wnt pathway activation is crucial for the initiation of new bone formation,^19^ while its dysregulation can lead to abnormal bone remodelling and even tumour formation.^20^, ^21^ On the other hand, non‐canonical Wnt ligands and downstream signals, independent of β‐catenin, also play a significant role in bone formation and resorption.^22^ *Wnt5a*, a key member of the non‐canonical Wnt signalling pathway, has been shown to enhance the proliferation and differentiation potential of BMSCs, and promote osteoblast maturation.^23^ Studies have demonstrated that mice with osteoblast‐specific *Wnt5a* knockout exhibit reduced bone mass, while treatment with exogenous *Wnt5a* leads to improved osteogenic outcomes.^24^, ^25^ However, conflicting findings by Hasegawa et al.^26^ suggested that activation of the non‐canonical Wnt pathway may actually inhibit osteogenic differentiation of stem cells. Mechanistically, Wnt5a can interact with various receptors in response to different cellular environments, activating either canonical or non‐canonical Wnt signalling pathways, thereby playing a dual role in bone homeostasis regulation.^27^ Collectively, Wnt5a is recognized as an essential factor for osteogenesis, but its specific role in OP remains unclear, with more research needed to uncover its intricacies.

In order to comprehensively investigate the bone‐forming potential of OP‐ASCs and uncover the underlying regulatory mechanisms, an osteoporotic mouse model was initially established through bilateral ovariectomy. Subsequently, OP‐ASCs were extracted from the adipose tissue in the groin area of these mice, revealing a significant decrease in their ability to form bone. Then, the key regulators within the Wnt signalling pathway were screened by western blot, polymerase chain reaction (PCR) and whole‐genome sequencing. Consequently, *Ctnnb1* and *Fzd6* were identified as exhibiting low expression levels in the OP, while *Gsk‐3β* and *Wnt5a* demonstrated aberrantly elevated expression. We then evaluated the impact of these regulators on the bone‐forming ability of OP‐ASCs through various modulations, including gene knockdown and overexpression within the Wnt pathway. Furthermore, the effect of Wnt5a on OP‐ASCs' osteogenic abilities was further validated in vivo by constructing a standard mouse cranial defect model. Collectively, these findings illuminate a novel mechanism governing the regulation of OP, with significant implications for how bone defects in OP can be treated at a molecular level.

## 2 MATERIALS AND EXPERIMENTAL PROCEDURE

### 2.1 Establishment of osteoporotic mice

To initiate, 18 female C57BL/6 mice (6 weeks) were purchased form Chong Qing Teng Xin Biological Technology Co. Ltd, (Chong Qing, China), and we divide them into two groups: an ovariectomy group (OVX) and a control group (Con). The OVX mice underwent an intraperitoneal injection of 1% pentobarbital sodium (30 mg/kg) for anaesthesia, followed by the surgical removal of bilateral ovaries to induce OP, as previous.^28^ In the control group, adipose tissues equivalent to the weight of the ovaries were removed instead. The experiments were carried out in compliance with the National Institutes of Health (NIH) guidelines for the Care and Use of Laboratory Animals and were approved by the Ethics Committee of Southwest Medical University.

### 2.2 Micro‐computed tomographic analysis

Six weeks post‐operation, we conducted a micro‐CT analysis of the femurs from both OVX and Con mice, using a GE Healthcare system. This analysis examined differences in bone volume fraction (BV/TV, %), trabecular number (Tb. N) and trabecular space (Tb. Sp) using the sophisticated Mimics 10.01 software for data analysis.

### 2.3 Histological examination

Following micro‐CT scanning, the bone samples underwent a 3‐week decalcification in 10% EDTA. Post‐decalcification, the samples were paraffin‐embedded, sectioned, and deparaffinized for haematoxylin and eosin (H&E) staining, the slides underwent rehydration, and staining with haematoxylin for 5 min, followed by eosin for 2 min. Alternatively, for Masson staining, the slides were exposed to Masson stain solution for 5 min, 5% phosphotungstate for 5 min and bright green staining solution for 5 min. Finally, femur structures were examined and captured using a microscope.

### 2.4 Extraction and cultivation of ASCs and OP‐ASCs

ASCs and OP‐ASCs were harvested from adipose tissue in the inguinal region of Con and OVX mice (aged 14–15 weeks) respectively and treated with collagenase. The resulting cell suspensions were cultured in flask, with non‐adherent cells being washed away after 48 h. The adherent cells were then cultured in complete medium, with regular medium changes and passaging at 80% confluence.

### 2.5 Cell surface antigen analysis

We prepared cell suspensions of ASCs and OP‐ASCs (5 × 10^6^ cells/mL), staining them with specific fluorescent dye‐labelled monoclonal antibodies against CD14, CD34, CD44, CD90, CD105 and Sca‐1. Post‐incubation, cells were analysed using BD's FACSCalibur flow cytometer, with data processed through WinMDI2.8 software.

### 2.6 Osteogenic and adipogenic differentiation assessment

ASCs and OP‐ASCs were seeded in 6‐well plates for differentiation studies. Osteogenic differentiation was induced by using Cyagen's osteogenic medium, with ALP staining at 14 days and alizarin red staining at 21 days.

For adipogenic differentiation, cells were cultured in Cyagen's adipogenic medium, involved culturing the cells in solution A for 3 days, followed by a single day in solution B. After five cycles of alternating between solutions A and B, the cells were then kept in solution B for an additional 5 days until lipid droplets became large and round enough. The formation of lipid droplets was then evaluated using Oil Red O staining.

### 2.7 qRT‐PCR

The cells were subjected to RNA extraction using the TRIzol reagent following the manufacturer's instructions. Subsequently, the PrimeScript RT reagent kit was utilized for cDNA synthesis. Real‐time PCR was performed on an ABI 7300 system with the SYBR Premix ExTaq kit. *Gpadh* was utilized as a reference for gene expression analysis, and the specific primer details can be found in Table S1.

### 2.8 Western blot

We adhered to the classic western blot technique, using specific antibodies for β‐Catenin (1:3000, ab32572), GSK‐3β (1:3000, ab32391), FZD6 (1:1000, ab128916), WNT5a (1:1000, ab174100), OPN (1:3000, ab195541) and RUNX2 (1:1000, ab92336), and GAPDH (1:5000, ab181602) as the internal control to evaluate protein expression levels.

### 2.9 Cell transfection and Wnt5a silencing/overexpression experiments

To silence *Wnt5a*, three unique shRNA sequences were designed to target mouse *Wnt5a* and then inserted into the pLKD‐CMV‐G&PR‐U6‐shRNA vector. The most effective shRNA was selected using PCR and Western blot analysis for further experiments.

To overexpress *Wnt5a*, the complete cDNA sequences of mouse *Wnt5a* (Gene ID: 009524) were cloned into a lentiviral vector (pLenti‐EF1a‐EGFP‐P2A‐Puro‐CMV‐MCS‐3Flag). A set of specific forward (5′‐CGCAAATGGGCGGTAGGCGTG‐3′) and reverse (5′‐CATAGCGTAAAAGGAGCAACA‐3′) primers were utilized in the experiment. Stable *Wnt5a*‐silenced or overexpressed OP‐ASCs were obtained after selection with puromycin (2 μg/mL) for 72 h, ready for subsequent experiments.

### 2.10 Transplantation of ASCs‐seeded BCP scaffolds in a calvariae bone critical defect models

The Biphasic calcium phosphate (BCP) scaffolds used in this study were provided by the Key Laboratory of Oral Biomedical Engineering at Sichuan University. The scaffolds were 4 mm in diameter and 2 mm in thickness, comprising 70% hydroxyapatite and 30% tricalcium phosphate, and exhibited a porosity ranging from 60% to 80% and an average pore diameter of 100–400 μm.

Prior to cell seeding, the scaffolds underwent autoclaving and were then incubated in α‐MEM medium for 24 h. Cell and scaffold constructs were prepared by seeding 200 μL OP‐ASCs at 1 × 10^5^/mL onto the surface of a BCP scaffold, followed by culture in osteogenic induction medium for 48 h.

A calvariae bone critical defect model was established using 36 OVX mice, post‐ovariectomy at 6 weeks, which underwent the creation of 4 mm diameter circular defects in their calvaria. These mice were divided into six groups, each receiving different graft materials, including: (1) a cell‐free BCP scaffold; (2) a BCP scaffold infused with OP‐ASCs; (3) a BCP scaffold populated with Wnt5a‐overexpressing OP‐ASCs; (4) a BCP scaffold with OP‐ASCs infected with a control lentivirus for Wnt5a overexpressing; (5) a BCP scaffold seeded with OP‐ASCs containing Wnt5a knockdown; and (6) a BCP scaffold containing OP‐ASCs transfected with a control lentivirus for Wnt5a knockdown. The implanted materials were harvested for micro‐CT analysis at 4, 8 and 12 weeks post‐operation, with histological assessment performed 12 weeks post‐implantation using H&E and Masson staining.

### 2.11 Statistical analysis

Data in this study was reported as mean ± standard deviation (SD). The statistical analysis involved the use of the unpaired two‐tailed Student's *t* tests for comparisons between two groups, and one‐way analysis of variance for comparisons between multiple groups. The software utilized for statistical analyses was SPSS 17.0, with a significance level set at *p* < 0.05.

## 3 RESULTS

### 3.1 Successful establishment of the osteoporotic mouse model

**FIGURE 1:**
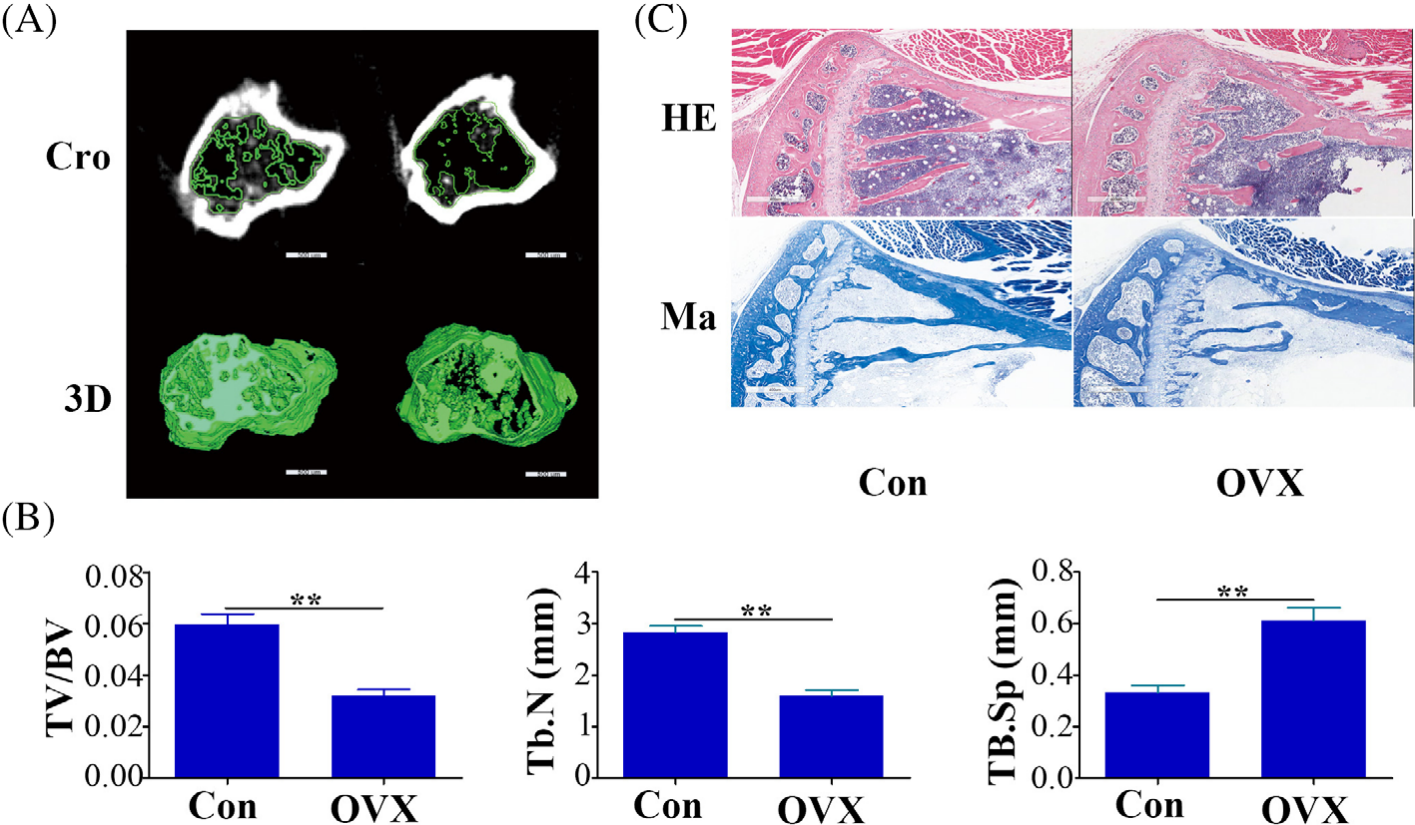
Successful construction of OP mice. (A) Micro‐CT scan findings of the femur in control and OVX mice, bar = 500 um. (B) The statistical analysis results of trabecular bone volume fraction (TV/BV), trabecular number (Tb. N) and trabecular separation (TB. Sp) based on the micro‐CT scans. (C) H&E and Masson staining results of the femur in control and OVX mice, bar = 400 um. Data are mean ± SD.**p < 0.01.

The osteoporosis mouse model was validated using micro‐CT analysis. Figure 1A illustrates noticeable differences in bone structure between the OVX group and Con group. Specifically, the OVX mice exhibited thinner cortical bone in the femur, enlarged bone marrow cavity and a disrupted trabeculae arrangement. Quantitative analysis confirmed these observations, showing a significant decrease in BV/TV and Tb. N, but an increase in Tb. Sp for the OVX group (Figure 1B). Histological staining further supported these findings, revealing significant structural changes in the femurs of OVX mice (Figure 1C).

### 3.2 OP‐ASCs demonstrate reduced osteogenic and increased adipogenic differentiation capabilities

**FIGURE 2:**
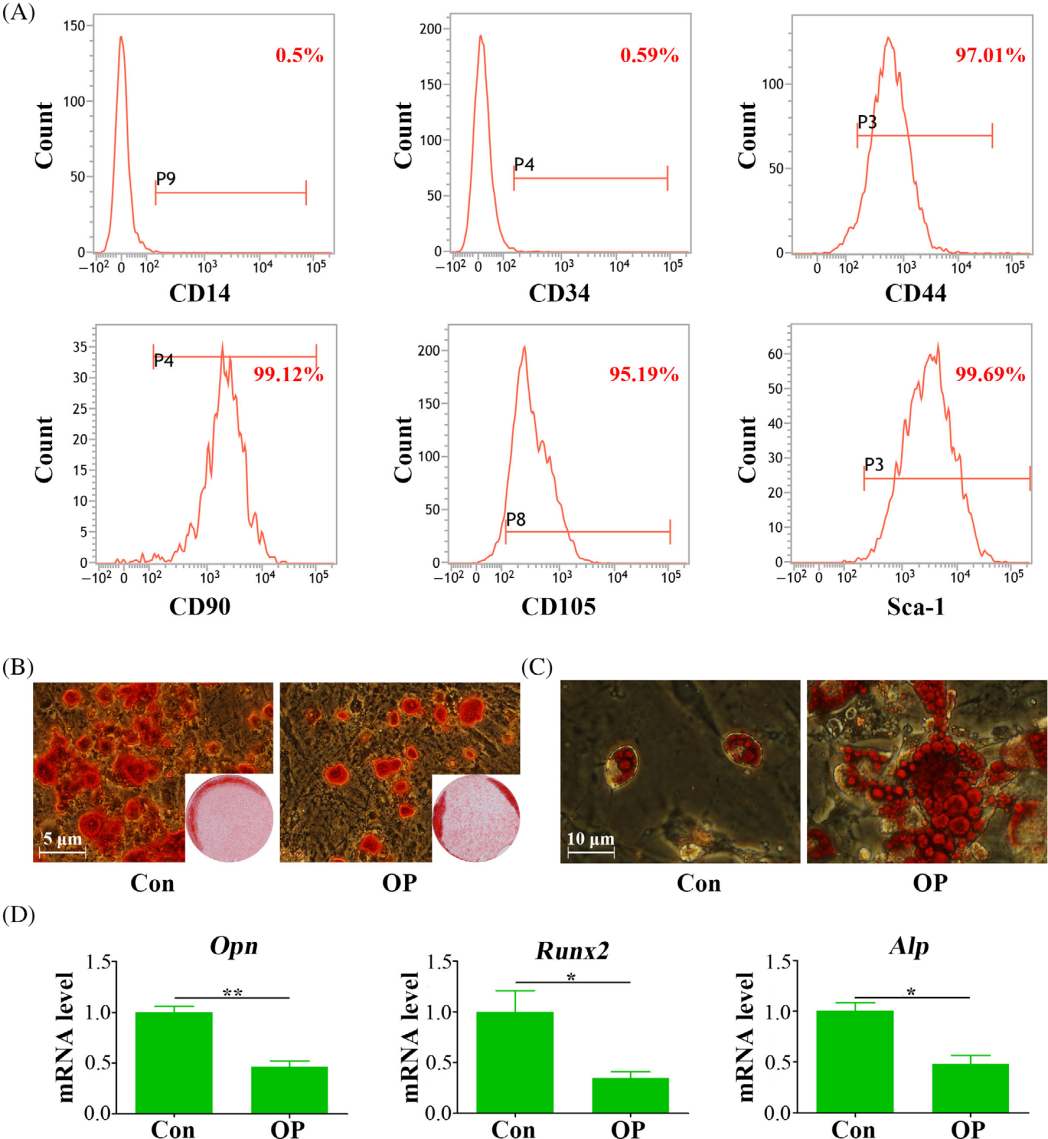
OP‐ASCs demonstrated diminished osteogenic differentiation capacity. (A) Flow cytometry analysis results of cells isolated from inguinal adipose tissue of OP mice. (B) Alizarin Red (ALR) staining results after 21 days of osteogenic induction in cells isolated from inguinal adipose tissue of control and OP mice. (C) Oil Red O staining results after 14 days of adipogenic induction in cells isolated from inguinal adipose tissue of control and OP mice. (D) qRT‐PCR analysis of osteogenic‐related factors (*Opn*, *Runx2*, *Alp*) expression levels in cells isolated from inguinal adipose tissue of control and OP mice. Data are mean ± SD. **p < 0.01.

Flow cytometric analysis confirmed the presence of mesenchymal markers CD44, CD90, CD105 and Sca‐1 on both ASCs and OP‐ASCs, along with the absence of CD14 and CD34 (Figure 2A). These findings provided definitive evidence supporting their characterization as mesenchymal stem cells.

Subsequent experiments focused on their potential for differentiation in multiple directions. Osteogenic induction over 21 days revealed that OP‐ASCs formed significantly fewer mineralized nodules than ASCs (Figure 2B). Moreover, qPCR analysis, performed subsequent to a three‐day period of osteogenic induction, demonstrated a marked reduction in the expression levels of gene pertinent to osteogenesis. This included *Alp*, *Runx2* and *Opn* in OP‐ASCs relative to ASCs, as illustrated in Figure 2D, suggesting a diminished osteogenic differentiation capacity. Conversely, after 21 days of adipogenic induction, OP‐ASCs displayed a noticeable increase in both the quantity and size of lipid droplets in comparison to ASCs (Figure 2C), suggesting an enhance ability for adipogenic differentiation.

### 3.3 Pivotal regulatory factors of osteoporosis were identified

**FIGURE 3:**
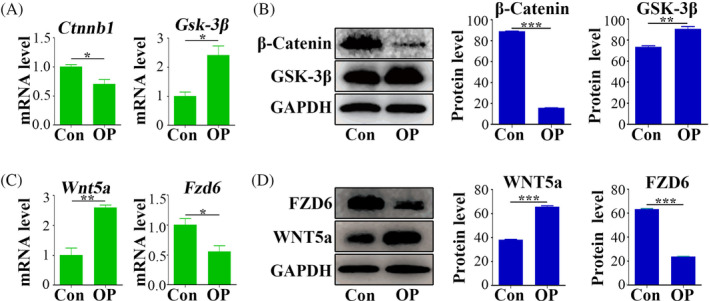
Inhibition of WNT signalling pathway in OP mice. (A) The transcriptional expression levels of *Ctnnb1* and *Gsk‐3β* in adipose tissues of OP and control mice. (B) The protein levels of β‐Catenin and GSK‐3β, along with their statistical analysis results in adipose tissues of OP and control mice. (C) The gene expression levels of *Wnt5a* and *Fzd6* in ASCs and OP‐ASCs. (D) The protein levels of β‐Catenin and GSK‐3β, along with their statistical analysis results in ASCs and OP‐ASCs. Data are mean ± SD. *p < 0.05; **p < 0.01.

This study assessed the protein and mRNA expression levels of β‐Catenin and Gsk‐3β expression levels in the adipose tissue of osteoporotic mice and control mice. qPCR and Western blot analyses indicated a marked reduction in *Ctnnb1* and β‐Catenin protein levels in osteoporotic mice (Figure 3A,B). Conversely, Gsk‐3β mRNA and protein showed higher expression in these mice compared to the control group (Figure 3A,B). Subsequently, gene chip analysis was conducted to discern Wnt‐related factors with substantial expression differences between OP‐ASCs and ASCs. These differences were confirmed by qPCR and Western blot, revealing an increase in Wnt5a mRNA and protein expression in OP‐ASCs, along with a decrease in Fzd6 mRNA and protein (Figure 3C,D).

### 3.4 The canonical Wnt pathway activation inhibited the non‐canonical Wnt pathway and enhanced osteogenic differentiation in OP‐ASCs

**FIGURE 4:**
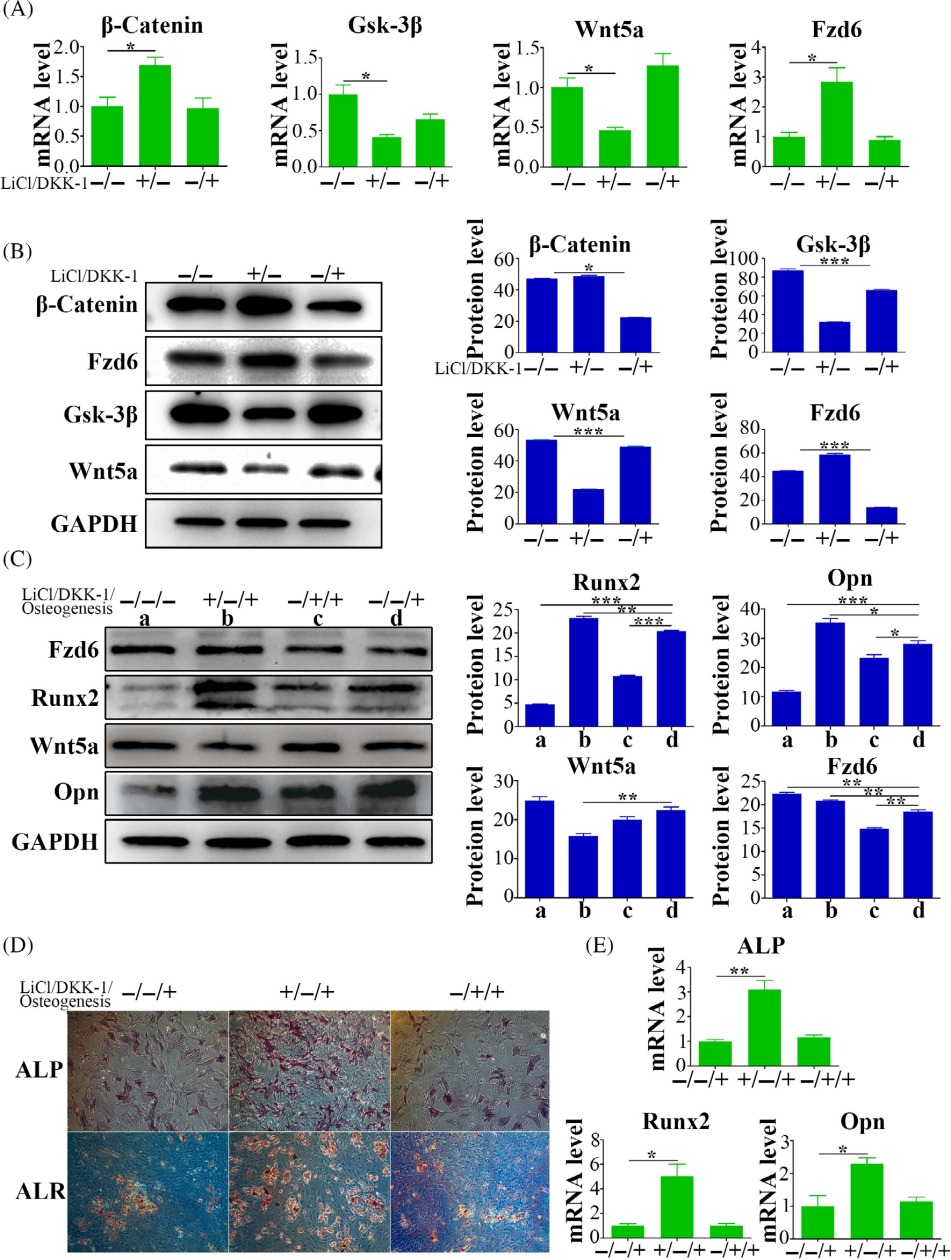
Activation of WNT signalling pathway suppresses non‐canonical WNT signalling and enhances osteogenic differentiation potential of OP‐ASCs. (A) The gene expression levels of *Ctnnb1*, *Gsk‐3β*, *Wnt5a* and *Fzd6* in OP‐ASCs following a 3‐day treatment with LiCl or DKK‐1. (B) The protein expression levels and statistical analysis in OP‐ASCs after a 3‐day treatment with LiCl or DKK‐1. (C) The protein expression levels and statistical analysis of WNT5a, FZD6, RUNX2 and OPN in OP‐ASCs subjected to simultaneous treatment with LiCl or DKK‐1 during a 3‐day osteogenic induction. (D) Staining results of ALP after 14 days and ALR after 21 days of osteogenic induction in OP‐ASCs treated simultaneously with LiCl or DKK‐1, bar = 50 um. (E) The gene expression levels of *Alp*, *Runx2* and *Opn* in OP‐ASCs after a 14‐day osteogenic induction with concurrent treatment of LiCl or DKK‐1. Data are mean ± SD. *p < 0.05; **p < 0.01; ***p < 0.001.

LiCl, a canonical Wnt pathway activator, resulted in upregulated *Ctnnb1* and *Fzd6* expression, and downregulated *Gsk‐3β* and *Wnt5a*. Intriguingly, in contrast to the control group, DKK‐1 treatment did not induce significant alterations in the expression of these genes (Figure 4A). However, at the protein level, LiCl treatment enhanced β‐Catenin and FZD6 expression accompanied by decreased expression of GSK‐3β and WNT5a. Conversely, DKK‐1 treatment reduced expression of β‐Catenin and FZD6, while promoting expression of GSK‐3β and WNT5a (Figure 4B).

Regarding osteogenesis, qPCR revealed elevated levels of osteogenic markers (*Alp*, *Runx2* and *Opn*) after LiCl treatment compared with levels in the control group. Conversely, DKK‐1 treatment did not elicit substantial differences in the expression levels of these genes (Figure 4E). Western blot substantiated the increased expression of RUNX2 and OPN following LiCl treatment, whereas their expression levels reduced by DKK‐1 treatment (Figure 4C). Additionally, ALP and ALR staining revealed distinct staining patterns and intensities between LiCl treatment and control groups, and between DKK‐1 treatment and control groups. Specifically, the LiCl treatment group exhibited more intense staining, whereas the DKK‐1 treatment group displayed weaker staining (Figure 4D).

### 3.5 Wnt5a inhibited osteogenic differentiation potential of OP‐ASCs

**FIGURE 5:**
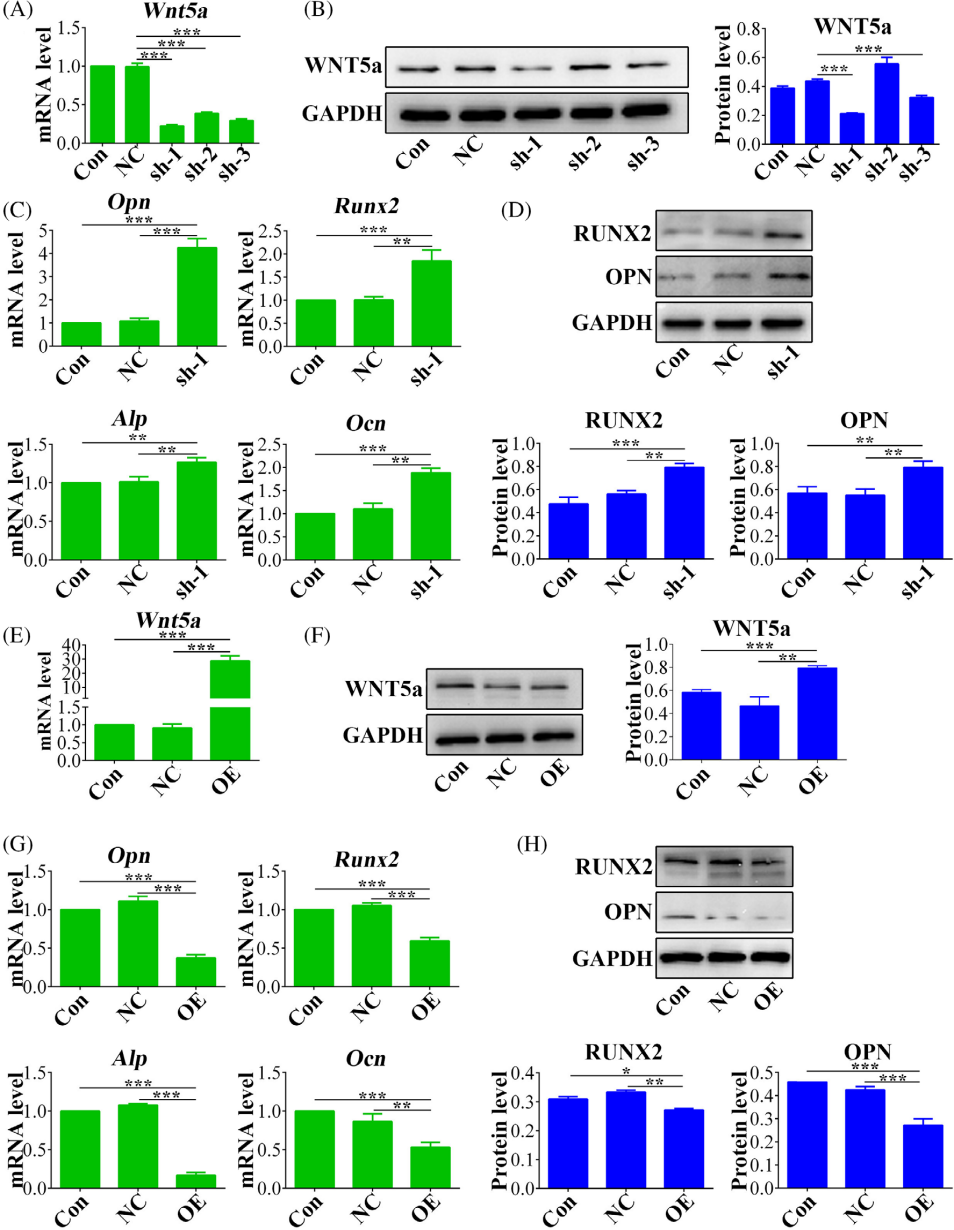
Wnt5a suppressed the osteogenic differentiation potential of OP‐ASCs in vitro. (A) Wnt5a knockdown effect was verified by qRT‐PCR and (B) western blot analysis. (C) The gene expression levels of *Opn*, *Runx2*, *Alp* and *Ocn* in OP‐ASCs after Wnt5a knockdown. (D) The protein expression levels of RUNX2 and OPN were assessed in OP‐ASCs after Wnt5a knockdown. (E) The upregulation of Wnt5a was validated at the mRNA level, and (F) protein level following OP‐ASCs infected with lentivirus containing *Wnt5a* cDNA. (G) The expression levels of *Opn*, *Runx2*, *Alp* and *Ocn* in OP‐ASCs were examined after Wnt5a overexpression. (H) The protein expression levels of RUNX2 and OPN were analysed in OP‐ASCs after Wnt5a overexpression. Data are mean ± SD. *p < 0.05; **p < 0.01; ***p < 0.001.

To explore the involvement of Wnt5a in osteoporosis, *Wnt5a* was knock down in OP‐ASCs using lentiviruses carrying *Wnt5a* shRNA sequences. The efficiency of silencing was confirmed at the mRNA level (Figure 5A) and also at the protein level (Figure 5B), and shRNA‐1 had the optimal silencing effect. Subsequent to knockdown, a significant reduction in mRNA expression levels of important osteogenic markers (*Ocn*, *Runx2*, *Opn*, *Alp*) was observed in the Wnt‐5a silenced OP‐ASCs compared with control groups (Figure 5C). Moreover, OPN and Runx2 protein expression levels were decreased in Wnt5a knockdown OP‐ASCs (Figure 5D). Conversely, Wnt5a overexpression was also induced using lentiviral vectors, resulting in higher levels of both Wnt5a mRNA (Figure 5E) and protein (Figure 5F). The upregulation resulted in elevated mRNA expression levels of the important osteogenic markers (Figure 5G), which was accompanied by corresponding decrease OPN and RUNX2 protein levels, compared to control groups comprising empty vectors (Figure 5H).

### 3.6 Wnt5a impeded the healing of osteoporotic bone defects in vivo

**FIGURE 6:**
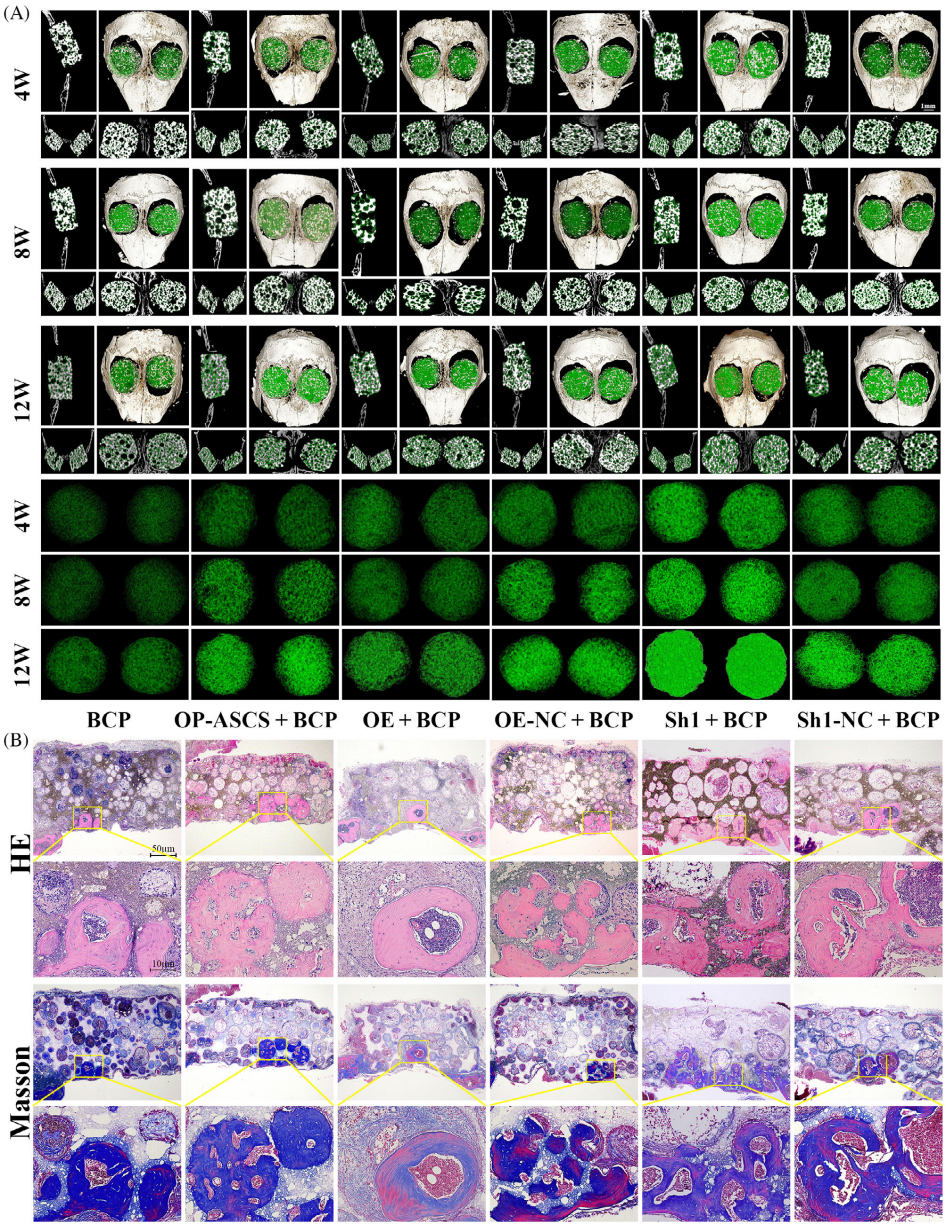
Wnt5a suppressed the bone regeneration capacity of OP‐ASCs in vivo. (A) Micro‐CT 3D reconstruction of bone regeneration in the critical sized calvarial bone defect transplanted with composite scaffold materials, including BCP, BCP + OP‐ASCs, BCP + OP‐ASCs overexpressing Wnt5a, BCP + OP‐ASCs with silenced Wnt5a and BCP + OP‐ASCs transfected with empty vectors silenced or overexpressed Wnt5a, after surgery for 4, 8 12 weeks, along with 3D reconstruction of the composite scaffold materials in each group of mice (lower panel). (B) H&E and Masson staining results of the composite scaffold material in each group of mice after surgery for 12 weeks. Data are mean ± SD. *p < 0.05; **p < 0.01; ***p < 0.001.

To examine Wnt5a's suppressive effects on bone formation in vivo, a transplantation experiment was carried out using constructs of OP‐ASCs and scaffolds. These constructs included BCP scaffolds along with OP‐ASCs that were either Wnt5a knockdown, Wnt5a‐overexpression or mock‐infected. Subsequently, these constructs were transplanted into mice with critical calvarial bone defects (Figure S1A). After 4, 8 and 12 weeks, micro‐CT and histological analysis were employed to assess bone regeneration. As shown in Figures 6A and S1B, the volume fraction and density of nascent bone formed in the Wnt5a knockdown group were significantly elevated relative to that in groups transplanted with cells or scaffolds alone, or OP‐ASCs transduced with control shRNA. Conversely, the volume fraction and density of newly formed bone in the Wnt5a overexpression group were considerably diminished compared with those in groups transplanted with cells or scaffolds alone, as well as the mock‐infected cells. Similar results were confirmed through H&E and Masson staining analyses (Figure 6B).

## 4 DISCUSSION

In the current study, we isolated ASCs from osteoporotic mice and observed a reduced capacity for osteogenic differentiation, companied by decreased expression levels of *Ctnnb1* and *Fzd6* within the canonical Wnt signalling pathway. Additionally, we noted an increase in the expression level of *Gsk‐3β* (the β‐catenin degrading enzyme) and an upregulation of *Wnt5a*, a component of the non‐canonical Wnt signalling pathway. It is important to highlight that both the canonical and non‐canonical Wnt signalling pathways are involved in regulating the osteogenic differentiation potential of OP‐ASCs. Furthermore, upon activation of the Wnt pathway using the Wnt signalling pathway activator LiCl, we observed a decrease in Wnt5a expression, a key factor in the non‐canonical Wnt pathway, which corresponded with an enhancement in the bone formation capacity of OP‐ASCs. Conversely, the application of the classical Wnt signalling pathway inhibitor DKK‐1 yielded opposing results. Through both in vitro and in vivo studies, we demonstrated that silencing Wnt5a enhanced the bone formation ability of OP‐ASCs. Our findings indicate that the canonical Wnt signalling pathway is suppressed while the non‐canonical Wnt pathway is activated in OP‐ASCs, suggesting a significant role of Wnt5a in osteoporotic development and its potential as a target for therapeutic intervention.

The Wnt signalling pathway has two main branches distinguished by the participation of β‐catenin: canonical and non‐canonical Wnt signalling pathway.^29^ In the canonical pathway, Wnt ligands bind to a receptor complex of frizzled proteins (Fzd) and coreceptors LRP5/6.^30^ In the absence of Wnts binding to this receptor complex, an intracellular destruction complex is created by Axin, APC, CK1 and GSK‐3β, resulting in the phosphorylation and breakdown of β‐catenin.^31^, ^32^, ^33^ Conversely, Wnt binding inhibits GSK‐3β expression, stabilizing and enabling nuclear translocation of β‐catenin, which then activates downstream genes related to Wnt signalling, promoting obsteoblast differentiation, proliferation and mineralization.^34^, ^35^, ^36^ Lithium affects the canonical pathway by reducing GSK‐3β activity,^37^ while DKK‐1 competitively inhibits this pathway by interacting with LRP5/6.^38^, ^39^ Mouse models have showed that the absence of DKK‐1 allele genes increases osteoblast numbers and bone formation, whereas specific overexpression of DKK‐1 in osteoblasts decreases bone mass and causes skeletal defects.^40^ The results of this study align with previously mentioned research, indicating that DKK‐1 inhibits the osteogenesis of OP‐ASCs by suppressing the canonical Wnt signalling pathway. Furthermore, this study found that DKK‐1 also upregulates the expression of Wnt5a, suggesting that DKK‐1 may inhibit osteogenesis through the activation of the non‐canonical Wnt signalling pathway. This observation is consistent with the work of Peng S et al.,^41^ which reported that DKK‐1 induces apoptosis through the activation of the non‐canonical Wnt and JNK signalling pathway.

Wnt5a as a non‐canonical Wnt ligand exerts a pivotal influence on the process of osteogenic differentiation in MSCs.^23^ Its expression is upregulated during the cellular differentiation, particular in osteoblasts.^24^ Knockout of the *Wnt5a* (*Wnt5a*−/−) in mouse models leads to perinatal death,^25^ while *Wnt5a*+/− mice show decreased trabecular bone mass, which is closely associated with diminished osteoblast counts and impaired bone formation rates.^42^ Wnt5a deficiency results in heightened β‐catenin pathway activity.^43^ Wnt5a inhibits the recruitment of activating factors to target gene promoter region, which competes with Wnt3a and associates with Fzd, thereby instigating ubiquitin ligase‐mediated breakdown of β‐catenin and effectively suppressing the canonical Wnt pathway.^44^ However, co‐expression of Wnt5a with Fzd5 or Fzd4 is a potent stimulus to activate the target genes and the subsequent β‐catenin cascade,^45^, ^46^ but this effect is only significant with receptors overexpression.^43^ This study also found that Wnt5a inhibits the osteogenic differentiation ability of OP‐ASCs, potentially through the suppression of the canonical Wnt signalling pathway and the activation of the non‐canonical Wnt signalling cascade. In contrast, Li et al.^47^ demonstrated that the synthetic presentation of a Wnt5a mimetic ligand activated non‐canonical Wnt signalling, elevated intracellular calcium levels, and promoted mechanotransduction and osteogenesis in human MSCs, and enhanced bone regeneration in vivo. Furthermore, Zhang et al.^48^ reported that the knockdown of Wnt5a attenuated osteogenic differentiation, while recombinant Wnt5a was able to rescue the suppressed osteogenic differentiation in human periodontal ligament cells (hPDLCs). The discrepancies in experimental results may be attributed to variations in ligand binding to Wnt5a, the distinct activation of the non‐canonical Wnt signalling pathway, and the differing experimental environments.

In the in vivo experiments, we also confirmed that the inhibition of Wnt5a resulted in a significant increase in the volume of newly formed bone in a well‐established mouse model of calvarial bone. Consequently, Wnt5a may be considered a potential target for the treatment of OP patients. However, this study utilized lentivirus to mediate Wnt5a silencing, which is not suitable for human application. Therefore, it is necessary to explore small molecule drugs that can mediate Wnt5a silencing to provide new methods or approaches for the treatment of OP. Additionally, the specific mechanism by which Wnt5a inhibits the canonical Wnt signalling pathway while activating the non‐canonical Wnt pathway need further investigation.

In conclusion, our findings elucidate that the canonical Wnt signalling pathway was downregulated, while the non‐canonical Wnt pathway was upregulated in OP‐ASCs. Moreover, the inhibition of Wnt5a emerges as a critical factor in increasing the osteogenic capabilities of OP‐ASCs both in laboratory settings and within living organisms. These discoveries indicate that targeting Wnt5a presents a therapeutic approach to improve bone formation for repairing bone fractures and defects in OP.

## AUTHOR CONTRIBUTIONS

LL designed, performed most experiments, analysed the experimental data and wrote the manuscript. SHL conducted culture of cells, collected and analysed the experimental data and wrote the manuscript. QML performed data analysis and manuscript editing. KH fed mice, conducted in vivo experiments and collected the experimental data. YJ performed project supervision. LZ performed data analysis. XRL performed data analysis and funding procurement. QL performed project supervision and manuscript editing. JGX conceived of, designed and supervised the study and revised the manuscript. All co‐authors have reviewed and approved this version of the manuscript.

## CONFLICT OF INTEREST STATEMENT

The authors declare no conflicts of interest.

## Supporting information


**Figure S1.** Wnt5a suppressed the bone regeneration capacity of OP‐ASCs in vivo. (A) Establishment of a critical sized calvarial bone defect in OP mice and implantation of composite scaffold materials in the model. (B) Quantification of the TV/BV of newly formed bone in the composite scaffold materials of each group of mice after surgery for 4, 8 and 12 weeks. Data are mean ± SD. **p < 0.01; ***p < 0.001.

**Table S1.** Primer sequences for qPCR.

## Data Availability

The data that support the findings of this study are available from the corresponding author upon reasonable request.

## References

[1] Huo S , Tang X , Chen W , et al. Epigenetic regulations of cellular senescence in osteoporosis. Ageing Res Rev. 2024;99:102235. 38367814 10.1016/j.arr.2024.102235

[2] Rachner TD , Khosla S , Hofbauer LC . Osteoporosis: now and the future. Lancet. 2011;377(9773):1276‐1287. 21450337 10.1016/S0140-6736(10)62349-5 PMC3555696

[3] Bai L , Feng MG , Zhang QW , et al. Synergistic osteogenic and anti‐apoptotic framework nucleic acid complexes prevent diabetic osteoporosis. Adv Funct Mater. 2024;34(28):2314789.

[4] Li Y , Cai ZW , Ma WJ , Bai L , Luo E , Lin Y . A DNA tetrahedron‐based Ferroptosis‐suppressing nanoparticle: superior delivery of curcumin and alleviation of diabetic osteoporosis. Bone Res. 2024;12(1):14. 38424439 10.1038/s41413-024-00319-7 PMC10904802

[5] Yeh EJ , Gitlin M , Sorio F , McCloskey E . Estimating the future clinical and economic benefits of improving osteoporosis diagnosis and treatment among postmenopausal women across eight European countries. Arch Osteoporos. 2023;18(1):68. 37191892 10.1007/s11657-023-01230-0 PMC10188417

[6] Zhao D , Cui W , Liu M , et al. Tetrahedral framework nucleic acid promotes the treatment of bisphosphonate‐related osteonecrosis of the jaws by promoting angiogenesis and M2 polarization. ACS Appl Mater Interfaces. 2020;12(40):44508‐44522. 32924430 10.1021/acsami.0c13839

[7] Morri M , Ambrosi E , Chiari P , et al. One‐year mortality after hip fracture surgery and prognostic factors: a prospective cohort study. Sci Rep. 2019;9(1):18718. 31822743 10.1038/s41598-019-55196-6 PMC6904473

[8] Khosla S , Hofbauer LC . Osteoporosis treatment: recent developments and ongoing challenges. Lancet Diabetes Endocrinol. 2017;5(11):898‐907. 28689769 10.1016/S2213-8587(17)30188-2 PMC5798872

[9] Prestwood KM , Pilbeam CC , Raisz LG . Treatment of osteoporosis. Annu Rev Med. 1995;46:249‐256. 7598461 10.1146/annurev.med.46.1.249

[10] Foessl I , Dimai HP , Obermayer‐Pietsch B . Long‐term and sequential treatment for osteoporosis. Nat Rev Endocrinol. 2023;19(9):520‐533. 37464088 10.1038/s41574-023-00866-9

[11] Reginster JY , Burlet N . Osteoporosis: a still increasing prevalence. Bone. 2006;38(2 Suppl 1):S4‐S9. 16455317 10.1016/j.bone.2005.11.024

[12] Aghebati‐Maleki L , Dolati S , Zandi R , et al. Prospect of mesenchymal stem cells in therapy of osteoporosis: a review. J Cell Physiol. 2019;234(6):8570‐8578. 30488448 10.1002/jcp.27833

[13] Zhao Y , Meng L , Zhang K , et al. Ultra‐small nanodots coated with oligopeptides providing highly negative charges to enhance osteogenic differentiation of hBMSCs better than osteogenic induction medium. Chin Chem Lett. 2020;32(1):266‐270.

[14] Chen TY , Xiao DX , Li YJ , et al. Tetrahedral framework nucleic acids regulate osteogenic differentiation potential of osteoporotic adipose‐derived stem cells. Chin Chem Lett. 2022;33(5):2517‐2521.

[15] Shao X , Hu Z , Zhan Y , Ma W , Quan L , Lin Y . MiR‐26a‐tetrahedral framework nucleic acids mediated osteogenesis of adipose‐derived mesenchymal stem cells. Cell Prolif. 2022;55(7):e13272. 35661456 10.1111/cpr.13272 PMC9251048

[16] Wu T , Tang H , Yang J , et al. METTL3‐m6 a methylase regulates the osteogenic potential of bone marrow mesenchymal stem cells in osteoporotic rats via the Wnt signalling pathway. Cell Prolif. 2022;55(5):e13234. 35470497 10.1111/cpr.13234 PMC9136513

[17] Canalis E . Wnt signalling in osteoporosis: mechanisms and novel therapeutic approaches. Nat Rev Endocrinol. 2013;9(10):575‐583. 23938284 10.1038/nrendo.2013.154

[18] Baron R , Kneissel M . WNT signaling in bone homeostasis and disease: from human mutations to treatments. Nat Med. 2013;19(2):179‐192. 23389618 10.1038/nm.3074

[19] Shen G , Ren H , Shang Q , et al. Foxf1 knockdown promotes BMSC osteogenesis in part by activating the Wnt/β‐catenin signalling pathway and prevents ovariectomy‐induced bone loss. EBioMedicine. 2020;52:102626. 31981979 10.1016/j.ebiom.2020.102626 PMC6992955

[20] Peng H , Jenkins ZA , White R , et al. An activating variant in CTNNB1 is associated with a sclerosing bone dysplasia and adrenocortical neoplasia. J Clin Endocrinol Metab. 2020;105(3):688‐695. 10.1210/clinem/dgaa034 31970420

[21] Deng Q , Li P , Che M , et al. Activation of hedgehog signaling in mesenchymal stem cells induces cartilage and bone tumor formation via Wnt/β‐catenin. Elife. 2019;8:e50208. 31482846 10.7554/eLife.50208 PMC6764825

[22] Keller KC , Ding H , Tieu R , Sparks NRL , Ehnes DD , zur Nieden NI . Wnt5a supports osteogenic lineage decisions in embryonic stem cells. Stem Cells Dev. 2016;25(13):1020‐1032. 26956615 10.1089/scd.2015.0367

[23] Santos A , Bakker AD , de Blieck‐Hogervorst JM , et al. WNT5A induces osteogenic differentiation of human adipose stem cells via rho‐associated kinase ROCK. Cytotherapy. 2010;12(7):924‐932. 20429785 10.3109/14653241003774011

[24] Maeda K , Kobayashi Y , Udagawa N , et al. Wnt5a‐Ror2 signaling between osteoblast‐lineage cells and osteoclast precursors enhances osteoclastogenesis. Nat Med. 2012;18(3):405‐412. 22344299 10.1038/nm.2653

[25] Oishi I , Suzuki H , Onishi N , et al. The receptor tyrosine kinase Ror2 is involved in non‐canonical Wnt5a/JNK signalling pathway. Genes Cells. 2003;8(7):645‐654. 12839624 10.1046/j.1365-2443.2003.00662.x

[26] Hasegawa D , Wada N , Yoshida S , et al. Wnt5a suppresses osteoblastic differentiation of human periodontal ligament stem cell‐like cells via Ror2/JNK signaling. J Cell Physiol. 2018;233(2):1752‐1762. 28681925 10.1002/jcp.26086

[27] Kumawat K , Gosens R . WNT‐5A: signaling and functions in health and disease. Cell Mol Life Sci. 2016;73(3):567‐587. 26514730 10.1007/s00018-015-2076-y PMC4713724

[28] Wang L , Huang C , Li Q , et al. Osteogenic differentiation potential of adipose‐derived stem cells from ovariectomized mice. Cell Prolif. 2017;50(2):e12328. 28090705 10.1111/cpr.12328 PMC6529141

[29] Milat F , Ng KW . Is Wnt signalling the final common pathway leading to bone formation? Mol Cell Endocrinol. 2009;310(1–2):52‐62. 19524639 10.1016/j.mce.2009.06.002

[30] He X , Semenov M , Tamai K , Zeng X . LDL receptor‐related proteins 5 and 6 in Wnt/beta‐catenin signaling: arrows point the way. Development. 2004;131(8):1663‐1677. 15084453 10.1242/dev.01117

[31] Nong J , Kang K , Shi Q , Zhu X , Tao Q , Chen YG . Phase separation of Axin organizes the β‐catenin destruction complex. J Cell Biol. 2021;220(4):e202012112. 33651074 10.1083/jcb.202012112 PMC7931644

[32] Zhang D , Ni QQ , Wang SY , et al. APC mutations disrupt β‐catenin destruction complex condensates organized by Axin phase separation. Cell Mol Life Sci. 2024;81(1):57. 38279052 10.1007/s00018-023-05068-0 PMC10817841

[33] Gybeľ T , Čada Š , Klementová D , et al. Splice variants of CK1α and CK1α‐like: comparative analysis of subcellular localization, kinase activity and function in the Wnt signaling pathway. J Biol Chem. 2024;300(7): 107407. 38796065 10.1016/j.jbc.2024.107407 PMC11255964

[34] Komiya Y , Habas R . Wnt signal transduction pathway. Organogenesis. 2008;4(2):68‐75. 19279717 10.4161/org.4.2.5851 PMC2634250

[35] Fröhlich J , Rose K , Hecht A . Transcriptional activity mediated by β‐CATENIN and TCF/LEF family members is completely dispensable for survival and propagation of multiple human colorectal cancer cell lines. Sci Rep. 2023;13(1):287. 36609428 10.1038/s41598-022-27261-0 PMC9822887

[36] Cho HH , Kim YJ , Kim SJ , et al. Endogenous Wnt signaling promotes proliferation and suppresses osteogenic differentiation in human adipose derived stromal cells. Tissue Eng. 2006;12(1):111‐121. 16499448 10.1089/ten.2006.12.111

[37] Gregory CA , Perry AS , Reyes E , Conley A , Gunn WG , Prockop DJ . Dkk‐1‐derived synthetic peptides and lithium chloride for the control and recovery of adult stem cells from bone marrow. J Biol Chem. 2005;280(3):2309‐2323. 15504735 10.1074/jbc.M406275200

[38] Li J , Sarosi I , Cattley RC , et al. Dkk1‐mediated inhibition of Wnt signaling in bone results in osteopenia. Bone. 2006;39(4):754‐766. 16730481 10.1016/j.bone.2006.03.017

[39] Sun JF , Gao Y , Yao YX , et al. Bone tissue engineering based on sustained release of MiR29c‐modified framework nucleic acids from an injectable hydrogel. Chem Eng J. 2024;487:150706.

[40] Morvan F , Boulukos K , Clément‐Lacroix P , et al. Deletion of a single allele of the Dkk1 gene leads to an increase in bone formation and bone mass. J Bone Miner Res. 2006;21(6):934‐945. 16753024 10.1359/jbmr.060311

[41] Peng S , Miao C , Li J , Fan X , Cao Y , Duan E . Dickkopf‐1 induced apoptosis in human placental choriocarcinoma is independent of canonical Wnt signaling. Biochem Biophys Res Commun. 2006;350(3):641‐647. 17026960 10.1016/j.bbrc.2006.09.087

[42] Roberts JL , Liu G , Paglia DN , et al. Deletion of Wnt5a in osteoclasts results in bone loss through decreased bone formation. Ann N Y Acad Sci. 2020;1463(1):45‐59. 31919867 10.1111/nyas.14293

[43] Mikels AJ , Nusse R . Purified Wnt5a protein activates or inhibits beta‐catenin‐TCF signaling depending on receptor context. PLoS Biol. 2006;4(4):e115. 16602827 10.1371/journal.pbio.0040115 PMC1420652

[44] Sato A , Yamamoto H , Sakane H , Koyama H , Kikuchi A . Wnt5a regulates distinct signalling pathways by binding to Frizzled2. EMBO J. 2010;29(1):41‐54. 19910923 10.1038/emboj.2009.322 PMC2808370

[45] He X , Saint‐Jeannet JP , Wang Y , Nathans J , Dawid I , Varmus H . A member of the frizzled protein family mediating axis induction by Wnt‐5A. Science. 1997;275(5306):1652‐1654. 9054360 10.1126/science.275.5306.1652

[46] Umbhauer M , Djiane A , Goisset C , et al. The C‐terminal cytoplasmic Lys‐thr‐X‐X‐X‐Trp motif in frizzled receptors mediates Wnt/beta‐catenin signalling. EMBO J. 2000;19(18):4944‐4954. 10990458 10.1093/emboj/19.18.4944 PMC314225

[47] Li R , Lin S , Zhu M , et al. Synthetic presentation of noncanonical Wnt5a motif promotes mechanosensing‐dependent differentiation of stem cells and regeneration. Sci Adv. 2019;5(10):eaaw3896. 31663014 10.1126/sciadv.aaw3896 PMC6795506

[48] Zhang X , Chang M , Wang B , Liu X , Zhang Z , Han G . YAP/WNT5A/FZD4 axis regulates osteogenic differentiation of human periodontal ligament cells under cyclic stretch. J Periodontal Res. 2023;58(5):907‐918. 37340863 10.1111/jre.13143

