# Supplementary figures and images for: Role of Wnt5a in modulation of osteoporotic adipose‐derived stem cells and osteogenesis
Source: Cell Prolif. 2024 Sep 17;58(2):e13747. doi: 10.1111/cpr.13747 (PMC11839189; doi:10.1111/cpr.13747)

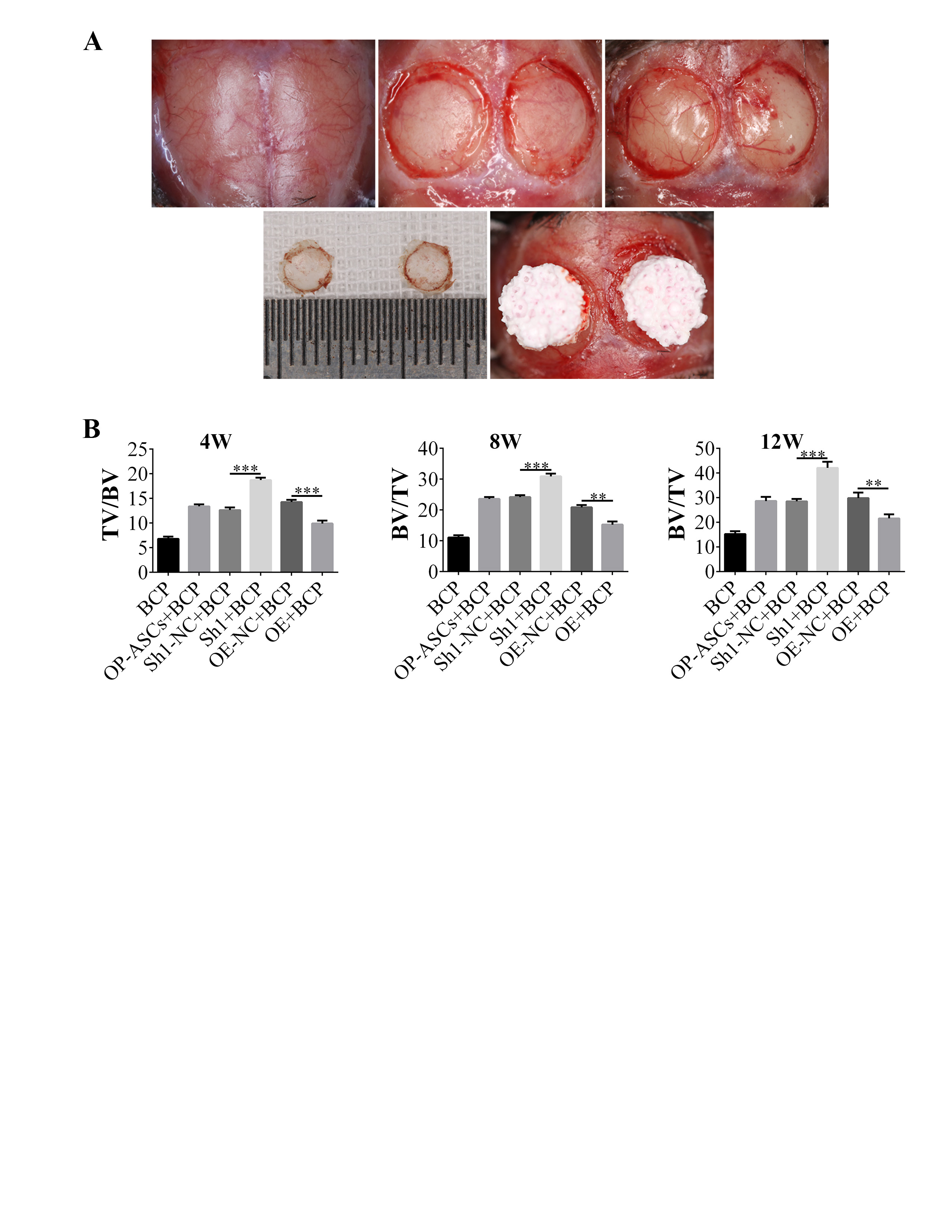

Supplement: Supplementary file 1 — Figure S1. Wnt5a suppressed the bone regeneration capacity of OP‐ASCs in vivo. (A) Establishment of a critical sized calvarial bone defect in OP mice and implantation of composite scaffold materials in the model. (B) Quantification of the TV/BV of newly formed bone in the composite scaffold materials of each group of mice after surgery for 4, 8 and 12 weeks. Data are mean ± SD. **p < 0.01; ***p < 0.001. [file CPR-58-e13747-s002.jpg]
