# Supplementary material for: Role of Wnt5a in modulation of osteoporotic adipose‐derived stem cells and osteogenesis
Source: Cell Prolif. 2024 Sep 17;58(2):e13747. doi: 10.1111/cpr.13747 (PMC11839189; doi:10.1111/cpr.13747)
Supplement: Supplementary file 2 — Table S1. Primer sequences for qPCR. [file CPR-58-e13747-s001.docx]

**Table S1 Primer Sequences for qPCR**

| **Genes** | **GenBank accession no.** | **Primer sequence 3’-5’** |
| --- | --- | --- |
| *GAPDH* | NM_008084 | F:ACAACTTTGGTATCGTGGAAGG |
|  |  | R:GCCATCACGCCACAGTTTC |
| *β-Catenin* | NM_001079520 | F:TGTGAATCCCAAGTACCAGTGT |
|  |  | R:CGTCAGACAAAGGAGAAACATT |
| *Gsk-3β* | NM_019827 | F:AGGCTGTGTGTTGGCTGAAT |
|  |  | R:TTTGCTCCCTTGTTGGTGTT |
| *Wnt5a* | NM_001256224 | F:AGCCCAGCTGATTCTTAATACC |
|  |  | R:GCTCAACTACATGGGACTTTCT |
| *Fzd6* | NM_008056 | F:ATGGAAAGGTCCCCGTTTCTG |
|  |  | R:GGGAAGAACGTCATGTTGTAAGT |
| *ALP* | NM_007431 | F: ACACCTTGACTGTGGTTACTG |
|  |  | R:CCATATAGGATGGCCGTGAAG |
| *Runx2* | NM_001015051 | F:CCGCCTCAGTGATTTAGGGC |
|  |  | R:GGGTCTGTAATCTGACTCTGTCC |
| *OPN* | NM_001251830 | F:CTCCATTGACTCGAACGACTC |
|  |  | R:CAGGTCTGCGAAACTTCTTAGAT |
| *OCN* | NM_001037939.1 | F:CCATCTTTCTGCTCACTCTGC |
|  |  | R:ACCTTATTGCCCTCCTGCTT |
